# Dietary fructose and its association with the metabolic syndrome in Lebanese healthy adults: a cross-sectional study

**DOI:** 10.1186/s13098-022-00800-5

**Published:** 2022-02-09

**Authors:** Rita Aoun, Fatima Al Zahraa Chokor, Mandy Taktouk, Mona Nasrallah, Hussain Ismaeel, Hani Tamim, Lara Nasreddine

**Affiliations:** 1 Department of Nutrition and Food Sciences, Faculty of Agricultural and Food Sciences, American University of Beirut, Beirut, Lebanon; 2 Department of Internal Medicine, Division of Endocrinology, Faculty of Medicine, American University of Beirut Medical Center, Beirut, Lebanon; 3 Vascular Medicine Program, American University of Beirut Medical Center, Beirut, Lebanon; 4 Division of Cardiology, Department of Internal Medicine, Faculty of Medicine, American University of Beirut Medical Center, Beirut, Lebanon; 5 Department of Internal Medicine, Faculty of Medicine, American University of Beirut Medical Center, Beirut, Lebanon; 6 Clinical Research Institute, Faculty of Medicine, American University of Beirut Medical Center, Beirut, Lebanon

**Keywords:** Metabolic syndrome, Dietary fructose, Natural fructose, Added fructose, Cross-sectional study, Adults, Lebanon

## Abstract

**Background:**

Epidemiological studies investigating the association between dietary fructose intake and the metabolic syndrome (MetS) are scarce and have produced controversial findings. This study aimed at (1) assessing total dietary fructose intake in a sample of Lebanese healthy adults, and determining the intake levels of natural vs. added fructose; (2) investigating the association of dietary fructose with MetS; and (3) identifying the socioeconomic and lifestyle factors associated with high fructose intake.

**Methods:**

A cross-sectional survey was conducted on a representative sample of adults living in Beirut, Lebanon (n = 283). Anthropometric and biochemical data were collected, and dietary intake was assessed using a food frequency questionnaire. Intakes of naturally-occurring fructose from fructose-containing food sources, such as fruits, vegetables, honey, were considered as “natural fructose”. Acknowledging that the most common form of added sugar in commodities is sucrose or High Fructose Corn Syrup (HFCS), 50% of added sugar in food products was considered as added fructose. Total dietary fructose intake was calculated by summing up natural and added fructose intakes. Logistic regression analyses were conducted to investigate the association of total, added and natural fructose intakes with the MetS and to identify the socioeconomic predictors of high fructose intake.

**Results:**

Mean intake of total fructose was estimated at 51.42 ± 35.54 g/day, representing 6.58 ± 3.71% of energy intakes (EI). Natural and added fructose intakes were estimated at 12.29 ± 8.57 and 39.12 ± 34.10 g/day (1.78 ± 1.41% EI and 4.80 ± 3.56% EI), respectively. Participants in the highest quartile of total and added fructose intakes had higher odds of MetS (OR = 2.84, 95%CI: 1.01, 7.94 and OR = 3.18, 95%CI: 1.06, 9.49, respectively). In contrast, natural fructose intake was not associated with MetS. Age, gender and crowding index were identified as factors that may modulate dietary fructose intakes.

**Conclusions:**

The observed association between high added fructose intake and the MetS highlights the need for public health strategies aimed at limiting sugar intake from industrialized foods and promoting healthier dietary patterns in Lebanon.

## Background

Fructose, the sweetest tasting carbohydrate (CHO), is consumed in significant amounts in the human diet. It occurs naturally in fresh fruits, vegetables and honey, with its traditional consumption ranging between 16 and 20 g/day. However, over the past 40 years, the intake of processed foods and beverages that are sweetened with High Fructose Corn Syrup (HFCS) or sucrose has escalated dramatically, resulting in significant increases in dietary fructose intake, to levels reaching as high as 85–100 g/day [1]. Temporal trend investigations have suggested parallel increasing trends in the prevalence of obesity, type 2 diabetes and cardiovascular diseases around the globe [2–5]. It was postulated that the metabolism of dietary fructose increases the risk of the metabolic syndrome (MetS) [2, 6], a constellation of cardiometabolic risk factors including insulin resistance, elevated blood pressure (BP), impaired glucose tolerance, hyperglycemia, atherogenic dyslipidemia (hypertriglyceridemia coupled with low high-density lipoprotein cholesterol (HDL-C) levels) and central adiposity [7, 8]. In turn, the MetS increases the risk for developing type 2 diabetes, cardiovascular diseases and all-cause mortality [9, 10]. A longitudinal study of 2902 adults followed for 11 years, showed that participants with the MetS had an adjusted relative risk for type 2 diabetes of 10.3 (5.44–19.5) and for cardiovascular diseases of 2.13 (1.43–3.18) [11].

Animal experiments and human clinical studies have investigated some of the mechanistic links between high fructose intake and the MetS, and suggested that excessive fructose intake may activate lipogenesis, induce insulin resistance and increase the risk of hypertension [12–20]. Fructose metabolism may also stimulate uric acid production leading to hyperuricemia [21], an independent risk factor for many pathological conditions, including the MetS [22]. However, in many instances, these studies have been criticized for using unrealistically high amounts of pure fructose [17, 23, 24]. Evidence from epidemiological studies that investigated current amounts of dietary fructose consumption and their association with the MetS are scarce and have produced controversial findings [25–28]. Indeed, an analysis of the National Health and Nutrition Examination Survey (NHANES) 1999–2006 database indicated that fructose ordinary consumption in the American diet was not positively associated with indicators of MetS [25]. On the other hand, other epidemiological studies have indicated a positive association of fructose consumption with cardiometabolic abnormalities [27, 28]. In some other studies, the association between fructose intake and cardiometabolic risk factors lost significance when the analysis was adjusted for body weight [29], suggesting that obesity may be responsible for the development of these cardiometabolic factors rather than fructose intake. For instance, an analysis of healthy male adults (n = 40,389) from the longitudinal Health Professionals Follow-Up Study, showed that the significant association between sugar-sweetened beverage (SSB) consumption and type 2 diabetes lost its significance after adjustment for confounders, which included body mass index (BMI) [29, 30]. The discrepancy in findings may also result from the fact that many of the available epidemiological studies have assessed the association between MetS and total fructose intake, without any differentiation between naturally occurring vs. added fructose intakes. Dietary sources of naturally occurring fructose include fruits and vegetables that are rich in antioxidants, phytochemicals, fiber, minerals and vitamins, and which are individually and synergistically beneficial to cardiometabolic health [1, 25]. In contrast, the sources of added fructose are mostly high sugar, energy-dense processed foods that were linked to increased cardiometabolic risk [1, 14, 25].

The controversy in the available findings highlights the need for more research examining dietary fructose intake as a cardiometabolic risk factor in epidemiological settings, while also distinguishing between naturally occurring vs. added fructose intakes. To date, the association between dietary fructose and the MetS has not been investigated in the Arab Eastern Mediterranean Region (EMR), a region that is witnessing the nutrition transition with its characteristic shifts in diet, lifestyle and body composition [31]. The region harbors one of the highest rates of obesity and the MetS worldwide [32], while also witnessing significant increases in sugar intakes [33]. Lebanon, a small country of the EMR, is no exception with earlier studies reporting a high prevalence of the MetS (34.7%) [34] in the adult population, while data on dietary fructose intake are completely lacking. It is in this context that this study was conducted with the aim of (1) assessing total dietary fructose intake in a sample of Lebanese healthy adults, and determining the intake levels of natural vs. added fructose; (2) investigating the association of dietary fructose with the MetS in the study population; and (3) identifying the socioeconomic and lifestyle factors associated with high fructose intake.

## Methods

Data for the current study were obtained from the cross sectional survey “Assessment of Bisphenol A (BPA) levels and their association with the health status amongst the Lebanese population” that was conducted between March and May 2014 on a representative sample of Lebanese adults residing in the Greater area of Beirut. The survey protocol was approved by the Institutional Review Board of the American University of Beirut (AUB). All participants included in the study signed an informed consent form and had the right to withdraw from the study at any time.

### Study design and participants

The study design and sampling strategy are published elsewhere [35–37]. In brief, participants were selected using a multistage probability sampling of adults, where the strata were the districts in the Greater Beirut area. Within each district, neighborhoods then households were selected based on systematic random sampling. At the household level, when more than one participant was eligible, the interviewer chose the one with the most recent month of birth to participate. The inclusion criteria included (1) age above 18 years, (2) of Lebanese nationality, and (3) residing in Greater Beirut. Participants were excluded if they were on dialysis, mentally disabled or pregnant. Furthermore, given that the parent study aimed at assessing BPA exposure in the general population, subjects who reported to work in plastic or other chemical factories were excluded. A sample of 501 subjects participated in the parent survey. For the present study, the selection of participants was conducted according to the following criteria: (1) having complete dietary, anthropometric, BP and biochemical data and (2) healthy, with no previously known diagnosis of chronic disease or any metabolic abnormality. This yielded a sample of 314 participants. In addition, subjects who reported implausible energy intakes (EI) (i.e. < 500 or > 6000 kcal/day) were excluded from the study [38, 39]. Accordingly, 283 participants were included in the current analysis.

### Data collection

Subjects who agreed to participate in the study were invited to visit AUB for data collection, after an overnight fast. Data collection took place at the Department of Nutrition and Food Sciences (NFSC) in the Faculty of Agricultural and Food Sciences at AUB. The study participants were interviewed by trained nutritionists using a multicomponent questionnaire and a food frequency questionnaire (FFQ). Anthropometric measurements (weight, height and waist circumference (WC)) were also obtained and blood samples were drawn by licensed phlebotomists. The duration of the interview and data collection for each participant was approximately one hour.

#### Sociodemographic and lifestyle characteristics

The questionnaire inquired about the sociodemographic and lifestyle characteristics of the participants, including age (in years), gender, marital status (single or married), educational level (no schooling or primary school; intermediate school; secondary school or technical diploma; university degree) monthly household income (< 600$; between 600 and 2000$; > 2000$), smoking status (current smokers of cigarette or hookah; past smokers; non-smokers), crowding index (a proxy measure of socioeconomic status, calculated as the number of people living in a household per number of rooms available in the house—excluding the kitchen and bathrooms) [40] and physical activity level. The short version of the International Physical Activity Questionnaire (IPAQ) was adopted for physical activity assessment [41]. Accordingly, physical activity was categorized into 3 categories: low, moderate and high. Data about family and personal medical history of diseases were also obtained.

#### Anthropometric, BP and biochemical measurements

Anthropometric measurements of the study participants were obtained by trained personnel and according to standardized procedures [42]. Weight was measured to the nearest 0.1 kg using a calibrated electronic weighing scale (Inbody 3.0, Biospace Co. Ltd, Korea), while the subjects were wearing light clothes without shoes. Height was measured using a portable stadiometer (Seca 213, Germany) and recorded to the nearest 0.5 cm. The candidates were in a standing position, flat against the measuring board without shoes. BMI was calculated as weight (kg) divided by square of the height (meters) [43]. WC was measured to the nearest 0.5 cm, at the umbilical level, using an unstretched tape meter (Seca 201, Germany). The tape was placed around the abdomen without exerting any pressure on the skin. Body fat was assessed using the Bioelectrical Impedance Analysis technique (Inbody 3.0, Biospace Co. Ltd, Alpha-Tec s.a.r.l.).

After blood withdrawal, fasting blood glucose (FBG) levels were measured using an enzymatic method (Cobas 6000, Roche, Indianapolis, IN, USA). Levels of serum triglycerides (TG), HDL-C and low-density lipoprotein cholesterol (LDL-C), were measured using an enzymatic spectrophotometric technique using Vitros 350 analyzer (Ortho-Clinical Diagnostics, Johnson and Johnson, 50–100 Holmers Farm Way, High Wycombe, Buckinghamshire, HP12 4DP, United Kingdom). Sitting BP was measured after a ten-minute rest using a standard digital sphygmanometer. All measurements were taken twice and the average of the two values was used.

MetS was defined based on the harmonized definition of the International Diabetes Federation (IDF). Participants were classified as having the MetS if they had at least 3 out of 5 of the following metabolic abnormalities: (1) Abdominal obesity: WC ≥ 94 cm for men and WC ≥ 80 cm for women (Eastern Mediterranean and Middle Eastern populations are recommended to use European data), (2) elevated BP: ≥ 130 mmHg systolic or ≥ 85 mmHg diastolic, (3) elevated fasting blood sugar ≥ 100 mg/dL (5.6 mmol/L), (4) elevated TG ≥ 150 mg/dL (> 1.69 mmol/L), (5) low serum HDL-C: < 40 mg/dL (< 1.04 mmol/L) for men and < 50 mg/dL (< 1.29 mmol/L) for women [7].

#### Dietary intake assessment and fructose intake estimation

Dietary data were collected using a semi-quantitative, culture specific FFQ with 86-food items [34, 36, 44]. The FFQ referred to the participant’s dietary intake during the past year, and subjects were asked to record the frequency of their food and beverage consumption per day, week, month, year or never. Participants were given the choice to estimate their intakes in grams or in terms of a reference portion size. Common household measures and a standard two-dimensional food portion visual chart [45] were used to assist participants in portion size estimation.

For data entry, a database application using Microsoft Access (Microsoft Corp., Redmond, WA, USA) was developed. Accordingly, for each beverage or food item, the frequency of consumption as reported by the participant was converted to daily intake. The Nutritionist Pro 1.2 software (Axxya Systems LLC, Stafford, TX, USA), and specifically the United States Department of Agriculture (USDA) database within the software, was used for energy and macronutrient content estimation. For traditional food items not included in the Nutritionist Pro database, recipes were added based on a local cookbook. Energy (kcal) was computed per gram for each food item/beverage on the FFQ list. The participant’s daily EI was then computed by summing the respective products of the quantity consumed and the energy per gram value for each food item/beverage [46]. The same procedure was used to calculate the daily intake of each macronutrient [47], as well as for total fructose.

There is no data on added vs. natural fructose in the database. Dietary intakes of naturally-occurring fructose from fructose-containing food sources, such as fruits, vegetables and honey, were considered as “natural fructose” [1]. Intakes of fructose from processed foods and beverages containing added sugar were defined as “added fructose”. Acknowledging that the most common form of added sugar in commodities is sucrose or HFCS, 50% of added sugar in food products was considered as added fructose [1, 25]. Eventually the intakes of total fructose were calculated by summing up natural fructose and added fructose consume [1].

### Statistical analysis

Frequencies, means and standard deviations were used to describe the various sociodemographic, lifestyle, anthropometric, biochemical and dietary characteristics of the overall study population and by MetS status. Categorical and continuous variables were compared between participants with and without MetS using Chi-square test and t-tests, respectively.

Logistic regression analyses were conducted to examine the association of dietary fructose intakes (total, added and natural) with the MetS, whereby the MetS was the dependent variable (yes/no) and fructose intake (total, added or natural) the independent variable. For the latter, intakes were categorized based on quartiles of intakes, Q4 being the highest level. Associations were investigated based on four models: (1) crude model; (2) model 1: adjusted for age and sex; (3) model 2: adjusted for age, sex, BMI, CHO (g/day), fibers (g/day), EI (kcal/day), smoking, education and physical activity level [1]; and (4) model 3: which is similar to model 2 but where we have replaced BMI by body fat. Results of logistic regression analyses were presented as odds ratio (OR) and 95% confidence interval (CI).

Multiple logistic regression analyses were also used to assess the sociodemographic and lifestyle predictors of high fructose intake (defined as Q4), using the sociodemographic and lifestyle characteristics as independent variables and high total, added or natural fructose intakes as the dependent variables. Statistical analysis was performed using the Statistical Analysis Package for Social Sciences (SPSS), version 24.0 (SPSS Inc., Chicago, IL, USA). In all the analyses, a p-value less than 0.05 was considered statistically significant.

## Results

Of the 283 study participants, 102 had the MetS according to the IDF definition, with an estimated prevalence of 36% [7]. The sociodemographic and lifestyle characteristics of the study population are presented in Table 1, according to MetS status. Participants with MetS were significantly older as compared with participants without MetS (44.8 ± 14.6 vs. 38.8 ± 12.7 years) (p < 0.001), with a significantly higher proportion of males presenting MetS as compared to those without MetS (45.1% vs. 25.4%) (p = 0.001). Significant differences in educational level were also observed between those having the MetS and those without (p = 0.01), whereby a lower proportion of participants with a university degree presented the MetS as compared to those without (4.9% vs. 18.9%). Other socioeconomic indicators such as income and crowding index did not differ between the two groups (Table 1). In addition, lifestyle factors including smoking and physical activity did not differ between groups (Table 1).

**Table 1:** Sociodemographic and lifestyle characteristics of the study sample (n = 283) ^†^

Variables^a^	All participants^b^ (n = 283)	Metabolic syndrome (n = 102)	No metabolic syndrome (n = 181)	P-value^c^
		Mean ± SD		
Age (years)	41.0 ± 13.7	44.8 ± 14.6	38.8 ± 12.7	** < 0.001**
	N (%)			
Sex				
Female	191 (67.5)	56 (54.9)	135 (74.6)	**0.001**
Male	92 (32.5)	46 (45.1)	46 (25.4)	
Marital Status				
Single^d^	90 (31.8)	36 (35.3)	54 (29.8)	0.344
Married	193 (68.2)	66 (64.7)	127 (70.2)	
Level of education				
No school or primary	89 (31.6)	38 (37.3)	51 (28.3)	**0.01**
Intermediate	77 (27.3)	31 (30.4)	46 (25.6)	
Secondary or technical	77 (27.3)	28 (27.5)	49 (27.2)	
University degree	39 (13.8)	5 (4.9)	34 (18.9)	
Income ($ per month)				
< 600	75 (28.4)	33 (33.0)	42 (25.6)	0.345
600 ≤ income ≤ 2000	165 (62.5)	60 (60.0)	105 (64.0)	
> 2000	24 (9.1)	7 (7.0)	17 (10.4)	
Smoking status^e^				
Never smoked	63 (22.3)	21 (20.6)	42 (23.2)	0.641
Current smoker	195 (68.9)	70 (68.6)	125 (69.1)	
Past smokers	25 (8.8)	11 (10.8)	14 (7.7)	
Physical Activity level				
Low intensity	131 (46.3)	51 (50.0)	80 (44.2)	0.199
Moderate intensity	88 (31.1)	34 (33.3)	54 (29.8)	
High intensity	64 (22.6)	17 (16.7)	47 (26.0)	
Crowding index				
≤ 1 person/room	109 (38.5)	45 (44.1)	64 (35.4)	0.146
> 1 person/room	174 (61.5)	57 (55.9)	117 (64.6)	

Bolded numbers are significant at p < 0.05

SD standard deviation

^a^Continuous variables are expressed as Mean ± SD and categorical variables are expressed as n (%)

^b^Lack of corresponding sum of frequencies with total sample size is due to missing data

^c^Significance was derived from chi-square for categorical variables and from independent t-test for continuous variables

^d^Single includes divorced, widowed and engaged

^e^Current smokers of either cigarette or narghile, past smokers of either cigarette or narghile

^†^Metabolic syndrome was defined according to Alberti et al. 2009 [7]

Table 2 presents the anthropometric, biochemical measurements and blood pressure data of the study sample (n = 283). The comparison of participants with and without MetS showed that those with MetS had significantly higher BMI (31.04 ± 5.40 vs. 26.37 ± 5.02 kg/m^2^), WC (100.65 ± 11.39 vs. 87.10 ± 12.02 cm) and body fat (32.45 ± 11.14 vs. 24.16 ± 10.08 kg) (p < 0.001). As expected, participants with MetS had significantly higher FBG (105.70 ± 17.71 vs. 93.99 ± 7.21 mg/dL) and TG levels (164.39 ± 79.96 vs. 96.07 ± 50.91 mg/dL), as well as significantly higher systolic (124.51 ± 18.42 vs. 111.71 ± 13.17 mmHg) and diastolic BP (77.41 ± 10.33 vs. 70.35 ± 8.16 mmHg), compared to participants without MetS (p < 0.001). The levels of LDL-C and HDL-C were also significantly different between the two groups (116.23 ± 38.25 vs. 101.78 ± 31.45 mg/dl for LDL-C and 42.87 ± 10.87 vs. 56.98 ± 16.04 mg/dl for HDL-C) (p < 0.001).

**Table 2:** Anthropometric characteristics, biochemical and blood pressure data of the study population (n = 283) ^†^

Variables^a^	All participants (n = 283)	Metabolic syndrome (n = 102)	No metabolic syndrome (n = 181)	p-value^b^
		Mean ± SD		
BMI (Kg/m^2^)	28.05 ± 5.62	31.04 ± 5.40	26.37 ± 5.02	** < 0.001**
Waist circumference (cm)	91.99 ± 13.46	100.65 ± 11.39	87.10 ± 12.02	** < 0.001**
Body fat (Kg)	27.15 ± 11.18	32.45 ± 11.14	24.16 ± 10.08	** < 0.001**
Triglycerides blood levels (mg/dL)	120.70 ± 70.88	164.39 ± 79.96	96.07 ± 50.91	** < 0.001**
HDL blood levels (mg/dL)	51.89 ± 15.89	42.87 ± 10.87	56.98 ± 16.04	** < 0.001**
LDL blood levels (mg/dL)	106.99 ± 34.69	116.23 ± 38.25	101.78 ± 31.45	** < 0.001**
Fasting blood glucose (mg/dL)	98.21 ± 13.31	105.70 ± 17.71	93.99 ± 7.21	** < 0.001**
Systolic blood pressure (mmHg)	116.68 ± 16.62	124.51 ± 18.42	111.71 ± 13.17	** < 0.001**
Diastolic blood pressure (mmHg)	72.89 ± 9.60	77.41 ± 10.33	70.35 ± 8.16	** < 0.001**

*BMI* body mass index, *HDL* high-density lipoprotein, *LDL* low-density lipoprotein, *SD* standard deviation

^a^ Data are expressed as Mean ± SD

^b^ Significance was derived from independent t-test

^†^ Metabolic syndrome was defined according to Alberti et al. 2009 [7]

Bolded numbers are significant at p < 0.05

Dietary intakes of the study participants are presented in Table 3. Overall, mean intake of total dietary fructose was 51.42 ± 35.54 g/day which represents 6.58 ± 3.71% of the total dietary energy intake (EI). Natural fructose intake was estimated at 12.29 ± 8.57 g/day, representing 1.78 ± 1.41% EI. The intake of added fructose was threefold higher than that of natural fructose, being estimated at 39.12 ± 34.10 g/day (4.80 ± 3.56% EI). The intakes of added fructose and total fructose were higher amongst those with the MetS (41.85 ± 33.70 and 53.69 ± 35.03 g/day, respectively) compared to those without (37.59 ± 34.31 and 50.14 ± 35.85 g/day, respectively), but the differences did not reach statistical significance (Table 3).

**Table 3:** Dietary energy and macronutrient intakes of the study population (n = 283) ^†^

Variables^a^	All participants (n = 283)	Metabolic syndrome (n = 102)	No metabolic syndrome (n = 181)	p-value^b^
		Mean ± SD		
Energy (Kcal/day)	3134 ± 1301	3232 ± 1337	3080 ± 1281	0.34
Carbohydrates (g/day)	388.22 ± 158.16	407.40 ± 170.21	377.41 ± 150.37	0.12
Carbohydrates (% of energy)	50.37 ± 8.30	50.98 ± 8.40	50.03 ± 8.25	0.36
Proteins (g/day)	102.83 ± 60.65	101.95 ± 50.44	103.30 ± 65.83	0.85
Proteins (% of energy)	13.02 ± 3.64	12.67 ± 3.22	13.22 ± 3.86	0.22
Fats (g/day)	131.81 ± 64.97	134.82 ± 67.28	130.12 ± 63.77	0.56
Fats (% of energy)	39.10 ± 7.86	38.56 ± 8.11	39.41 ± 7.72	0.38
Dietary fibers (g/day)	28.16 ± 11.78	28.70 ± 13.49	27.85 ± 10.72	0.56
Total sugar intake (g/day)	104.99 ± 58.45	111.18 ± 61.84	101.49 ± 56.32	0.18
Added fructose (g/day) ^c^	39.12 ± 34.10	41.85 ± 33.70	37.59 ± 34.31	0.31
Added fructose (% of Kcal/day)	4.80 ± 3.56	4.81 ± 2.97	4.79 ± 3.86	0.96
Natural fructose (g/day) ^d^	12.29 ± 8.57	11.84 ± 7.88	12.55 ± 8.95	0.51
Natural fructose (% of Kcal/day)	1.78 ± 1.41	1.61 ± 1.15	1.87 ± 1.53	0.12
Total fructose (g/day) (Added + Natural)	51.42 ± 35.54	53.69 ± 35.03	50.14 ± 35.85	0.42
Total fructose (% Kcal/day)	6.58 ± 3.71	6.42 ± 2.96	6.67 ± 4.07	0.59

*SD* standard deviation

^a^ Data are expressed as Mean ± SD

^b^ Significance was derived from independent t-test

^c^ Added fructose from industrialized foods and beverages containing beet or cane sugar/molasses, corn sweeteners and invert syrup

^d^ Natural fructose in fructose-containing food such as fruits, vegetables, honey

^†^ Metabolic syndrome was defined according to Alberti et al. 2009 [7]

Logistic regression analyses were performed to examine the association between fructose intake and the MetS (Table 4). Four models were performed: the crude model; model 1 adjusted for age and sex; model 2 adjusted for the variables included in model 1, in addition to BMI, CHO (g/day), fibers (g/day), EI (kcal/day), smoking (ex, current, never), education and physical activity level (yes, no) [1]; and model 3: which is similar to model 2, but where we have adjusted for body fat instead of BMI. Accordingly, the odds of MetS increased by 2.84 fold for participants in the highest quartile of total fructose intake (Q4) (OR = 2.84, 95%CI: 1.01, 7.94) after adjusting for BMI and other potential confounders (Model 2). Similarly, in the same model, high intake of added fructose (Q4) was associated with significantly higher odds of having the MetS (OR = 3.18, 95%CI: 1.06, 9.49) (Model 2). Significant associations were also found based on model 3 whereby the odds of MetS increased by 3.08 fold for participants in the highest quartile of total fructose intake (Q4) (OR = 3.08, 95%CI: 1.08, 8.79) and by 3.47 fold for participants in the highest quartile of added fructose intake (Q4) (OR = 3.47, 95%CI: 1.13, 10.59). However, no significant association was observed between natural fructose and the MetS.

**Table 4:** Associations between total, added and natural fructose intakes (quartiles, g/day) and MetS, based on logistic regression analyses

		Crude model^*^	Model 1^**^	Model 2^***^	Model 3^****^
			OR (95% CI)		
Total fructose intake (g/day)	Q1: < 26.74	Reference	Reference	Reference	Reference
	Q2: 26.75–41.99	0.66 (0.32, 1.34)	0.59 (0.27, 1.25)	1.02 (0.42, 2.43)	0.95 (0.40, 2.23)
	Q3: 41.99–64.05	0.79 (0.39, 1.58)	0.85 (0.40, 1.79)	1.09 (0.45, 2.68)	1.11 (0.45, 2.74)
	Q4: > 64.05	1.50 (0.76, 2.96)	1.87 (0.86, 4.02)	**2.84 (1.01, 7.94)**	**3.08 (1.08, 8.79)**
Added fructose intake (g/day)	Q1: < 15.10	Reference	Reference	Reference	Reference
	Q2: 15.10–28.40	0.82 (0.41, 1.66)	0.80 (0.38, 1.68)	1.30 (0.55, 3.04)	1.24 (0.52, 2.91)
	Q3: 28.41–51.48	0.88 (0.44, 1.76)	1.00 (0.47, 2.14)	1.33 (0.53, 3.34)	1.40 (0.56, 3.52)
	Q4: > 51.48	1.54 (0.78, 3.04)	2.10 (0.95, 4.63)	**3.18 (1.06, 9.49)**	**3.47 (1.13, 10.59)**
Natural fructose intake (g/day)	Q1: < 6.39	Reference	Reference	Reference	Reference
	Q2: 6.39–10.39	0.90 (0.45, 1.78)	0.88 (0.42, 1.81)	0.86 (0.38, 1.95)	0.91 (0.39, 2.07)
	Q3: 10.39–16.64	1.03 (0.52, 2.05)	0.96 (0.47, 1.97)	1.16 (0.49, 2.76)	1.06 (0.44, 2.50)
	Q4: > 16.64	0.88 (0.44, 1.76)	0.77 (0.37, 1.61)	1.04 (0.41, 2.64)	1.01 (0.39, 2.60)

*BMI* body mass index, *CHO* carbohydrates, *CI* confidence interval, *EI* energy intake, *OR* odds ratio, *MetS* metabolic syndrome, *Q* quartile

^*^ Crude model: No adjustments

^**^ Model 1: Adjusted for age and sex

^***^ Model 2: Adjusted for age, sex, BMI, CHO (g/day), fibers (g/day), EI (kcal/day), smoking, education and physical activity level

****Model 3: Adjusted for age, sex, body fat (Kg), CHO (g/day), fibers (g/day), EI (kcal/day), smoking, education and physical activity level

The association of sociodemographic and lifestyle characteristics with high intake of fructose (Q4) is shown in Table 5. The table displays crude ORs and adjusted ORs (AORs), after adjustment for variables that appeared to be significantly associated with fructose intake in the crude model. Based on the adjusted model, the odds of consuming high levels of natural fructose increased with age (OR = 1.02, 95%CI: 1.001, 1.042), while the odds of consuming high levels of added fructose decreased with age (OR = 0.95, 95%CI: 0.922, 0.973). Male gender was associated with a three-fold increase in the odds of consuming high levels of total and added fructose (OR = 2.90, 95%CI: 1.587, 5.301 and OR = 3.23, 95%CI: 1.713, 6.073 respectively). Higher crowding index, which is a reflection of lower socioeconomic status, was associated with lower odds of having high intakes of natural fructose.

**Table 5:** Demographic, socioeconomic and lifestyle correlates of high intakes of total, added and natural fructose intakes in Lebanese adults ^#^

	High total fructose intake Q4		High added fructose intake Q4		High natural fructose intake Q4	
	OR (95% CI)^	AOR (95% CI)*	OR (95% CI)^	AOR (95% CI)*	OR (95% CI)^	AOR (95% CI)*
Age (years)	**0.95 (0.929, 0.972)**	**0.96 (0.932, 0.980)**	**0.94 (0.922, 0.966)**	**0.95 (0.922, 0.973)**	**1.02 (1.003, 1.044)**	**1.02 (1.001, 1.042)**
Sex						
Female	Reference	Reference	Reference	Reference	Reference	–
Male	**3.22 (1.839, 5.643)**	**2.90 (1.587, 5.301)**	**3.49 (1.992, 6.137)**	**3.23 (1.713, 6.073)**	0.71 (0.393, 1.299)	–
Marital status						
Single	Reference	Reference	Reference	Reference	Reference	–
Married	**0.44 (0.254, 0.777)**	0.94 (0.473, 1.888)	**0.44 (0.254, 0.777)**	1.14 (0.544, 2.400)	1.47 (0.804, 2.706)	–
Level of education						
No school or primary	Reference	–	Reference	Reference	Reference	–
Intermediate	1.12 (0.530, 2.358)	–	1.20 (0.564, 2.552)	1.11 (0.489, 2.500)	0.86 (0.419, 1.777)	–
Secondary or technical	1.68 (0.825, 3.421)	–	**2.03 (0.999, 4.150)**	1.25 (0.564, 2.784)	0.86 (0.419, 1.777)	–
University degree	1.75 (0.746, 4.119)	–	1.66 (0.694, 3.992)	1.22 (0.451, 3.279)	1.71 (0.757, 3.843)	–
Income ($ per month)						
< 600	Reference	–	Reference	–	Reference	–
600 ≤ income ≤ 2000	1.27 (0.675, 2.426)	–	1.47 (0.769, 2.807)	–	0.82 (0.444, 1.525)	–
> 2000	1.13 (0.390, 3.317)	–	1.23 (0.419, 3.607)	–	0.85 (0.299, 2.456)	–
Smoking status						
Never smoked	Reference	–	Reference	–	Reference	–
Current smoker	1.90 (0.926, 3.915)	–	1.90 (0.926, 3.915)	–	1.11 (0.564, 2.191)	–
Past smokers	0.65 (0.164, 2.538)	–	0.64 (0.164, 2.538)	–	1.97 (0.717, 5.404)	–
Physical activity level						
Low intensity	Reference	–	Reference	–	Reference	–
Moderate intensity	1.36 (0.721, 2.576)	–	1.51 (0.802, 2.860)	–	1.14 (0.612, 2.129)	–
High intensity	1.75 (0.889, 3.446)	–	1.83 (0.929, 3.627)	–	1.07 (0.537, 2.154)	–
Crowding index						
≤ 1 person/room	Reference	–	Reference	–	Reference	Reference
> 1 person/room	1.38 (0.783, 2.444)	–	1.51 (0.849, 2.674)	–	**0.492 (0.285, 0.851)**	**0.517 (0.297, 0.899)**

*AOR* adjusted odds ratio, *CI* confidence interval, *OR* odds ratio, *Q* quartile

# Assessed using multiple logistic regression analysis.

^ ORs were presented with 95% CI using simple logistic regression.

* AORs were presented with 95% CI using multiple logistic regression analysis. The model was adjusted for sociodemographic characteristics found to be significant correlates of the dependent variable at the crude model.

## Discussion

This study is the first to characterize the dietary intake of fructose in the Arab Middle-East and to investigate the association of total, added and natural fructose intakes with the MetS. The study showed that Lebanese healthy adults consumed 51.4 g/day of total fructose, with the intake of added fructose being approximately three times higher than that of naturally occurring fructose (39.12 vs. 12.29 g/day). High intakes of total and added fructose were found to be significantly associated with higher odds of the MetS, whereas natural fructose intake did not increase the risk of the MetS in the study population. The study has also shed light on demographic and socioeconomic factors that may modulate dietary fructose intake, namely age, gender and crowding index.

Total dietary fructose intake as estimated in this study (51.42 ± 35.54 g/day), is similar to that reported from the US (48.07 ± 35.73 g/day) based on NHANES survey, while exceeding estimates reported from European countries, such as Germany and Finland, where total fructose intake ranged between 8.4 and 40.6 g/day [48, 49]. Total fructose intake in Lebanon also exceeds that reported from Iran, another Middle-Eastern country where dietary fructose intake was found to range between 37.3 ± 24.2 g/day in women and 46.5 ± 24.5 g/day in men [1]. Few studies have estimated added vs. natural fructose limiting the comparability of our findings. Added fructose intake amongst Lebanese adults (39.12 ± 34.10 g/day) exceeded the estimate reported from Iran (19.0–26.9 g/day) [1], while the consumption level of natural fructose in Lebanon (12.29 ± 8.57 g/day) was lower than that observed amongst Iranian adults (18.6–19.5 g/day). The high intake of added fructose (4.80 ± 3.56% kcal/day) compared to natural fructose (1.78 ± 1.41% kcal/day) in Lebanon may be a manifestation of the nutrition transition in the country. Indeed, Lebanon, like other countries of the Middle-East, is undergoing modernization and urbanization which resulted in significant changes in diet during the past few decades [50], away from the traditional diet towards a Westernized dietary pattern that is rich in processed and ultra-processed foods that are important sources of sugar, and particularly added fructose [51]. A recent study showed that the diet in Lebanon is shifting towards higher energy, higher sugar and lower intakes of fruits and vegetables [51]. Based on our study findings, added fructose represented 76% of total fructose intake and the latter was found to exceed the suggested upper limit of fructose intake (50 g/day) [52].

In our study, the prevalence of the MetS was estimated at 36%, which aligns with previous studies conducted amongst healthy adults in Lebanon (31.2–34.7%) [34, 53]. Our results showed a significant association between the intakes of total fructose and added fructose with the MetS, after adjustment for potential confounders. It should be emphasized that, the positive association was observed only in the fourth quartiles of fructose intake (> 64.05 g of total fructose/day and > 51.48 g of added fructose/day), which were associated with approximately a three-fold increase in the odds of having the MetS. The positive association between fructose consumption and increased risk of MetS is supported by several other studies [1, 3, 6, 54–56]. For instance, a previous cross-sectional study conducted in 2011 by Hosseini-Esfahani et al. in Iran, showed that men and women in the highest quartile of fructose intake, had 33% and 20% higher risk of developing MetS, respectively [1]. Similar results were documented in a systematic review and meta-analysis of clinical trials which also concluded that high fructose consumption from industrialized products is the main contributor to MetS [57]. In a meta-analysis of prospective cohorts by Malik et al., the pooled relative risk (RR) was 1.20 [95%CI: 1.02–1.42] comparing extreme quartile of SSBs intake, which is considered an important source of added fructose in the human diet [6]. An important mechanism that may explain this association between fructose intake and MetS is the altered appetite and satiety signals caused by high fructose intake which results in weight gain and visceral fat accumulation [58, 59]. For instance, fructose does not stimulate the production of insulin and leptin, two hormones known to play a role in the long-term regulation of appetite, food intake and energy expenditure [60]. On the other hand, fructose does not inhibit the secretion of ghrelin, a hormone that promotes hunger, and contributes to higher energy intake, which can subsequently lead to weight gain and body fact accumulation [61, 62]. Interestingly, in our study, the association of fructose with MetS became significant only after adjustment for confounders that included age, sex and BMI. The fact that the association became significant after adjustment for BMI or body fat, suggests that the association is driven by nutrient-specific mechanisms rather than merely excessive body weight or adiposity. These nutrient-specific mechanisms may include the unregulated hepatic fructose metabolism that causes hepatic lipid accumulation [63, 64], hyperuricemia [21, 65] and decreased insulin sensitivity [12], and hence MetS. A recent review by Legeza et al. (2017) also highlighted an interconnection between high fructose intake and enhanced generation of active glucocorticoids in the adipose tissue, the latter being mediated by increased activity of 11β-hydroxysteroid dehydrogenase 1 (11β-HSD1), which can also contribute to cardiometabolic abnormalities and the MetS [66]. Our study findings illustrating the high intake of added fructose in Lebanon coupled with its association with a three-fold increase in the odds of MetS is a public health concern, given its implications on the burden non-communicable diseases (NCDs) in the country. The MetS is in fact a risk factor for type 2 diabetes and cardiovascular diseases [67, 68], which are highly prevalent in Eastern Mediterranean countries, especially in Lebanon, and are assuming an escalating secular trend [69].

It is worth noting that in the current study no positive association was identified between natural fructose and MetS even after adjustment for potential confounders. The sources of natural fructose are principally fruits and vegetables which are rich in various bioactive compounds such as phytochemicals, antioxidants and fibers [70]. This wide array of protective nutrients may offset metabolic abnormalities of fructose and may prevent the development of obesity, diabetes and MetS [70]. Furthermore, small amounts of natural fructose coming from fruits and vegetables have been shown to improve hepatic glucose handling by increasing the uptake of glucose into the liver and glycogen deposition [71]. In fact, via its frucotose-1-phosphate metabolite, small amounts of fructose can activate the glucokinase enzyme, which promotes hepatic glucose uptake; fructose is also a potent glycogenic precursor that is converted to glycogen via the indirect pathway (i.e. from gluconeogenic precursors) [71–74].

In our study, age was associated with higher odds of having high consumption of natural fructose, while the opposite was observed for added fructose. The observed direct association between age and natural fructose intake is in line with previous studies suggesting that older adults tend to maintain traditional dietary habits and hence consume higher amounts of fruits and vegetables compared to younger ones, the latter typically having greater exposure to new and “fashionable” food products that are rich in added fructose [75, 76]. The age gradient in natural and added fructose intakes may also indicate a state of nutrition transition, from a traditional to a Western dietary pattern, a phenomenon that usually manifests itself in younger age groups as has been previously described in Lebanon and other countries of the Middle-East region [44, 51, 75, 77]. It is important to note that, despite age being associated with lower consumption of added fructose, a higher prevalence of the MetS was observed amongst older subjects in our study. The etiology of the MetS is in fact multifactorial, with age being recognized as a significant risk factor for the MetS [78]. However, our study showed that, after adjustment for potential confounders, which included age, high intakes of added fructose remained independent predictors of the MetS in the study sample.

In our study, male gender was associated with a three-fold increase in the odds of having high consumption of added fructose. These findings may be explained by the previously documented sex disparities in food consumption amongst Lebanese adults. For instance, a recent national study showed that Lebanese men had significantly higher intakes of soft drinks compared to women, while the latter had significantly higher intakes of fruits and vegetables [79]. Our study findings are also in line with previous studies conducted in Lebanon showing that males were more likely to adhere to the Western dietary pattern that is rich in added sugar (amongst other characteristics), while females had higher adherence to the Traditional Lebanese dietary pattern, that is richer in fruits and vegetables [36, 44, 80, 81]. Therefore, in addition to potential differences in sex-hormones and the predisposition for abdominal or visceral adiposity [82], sex-based disparities in dietary intakes and added sugar consumption may play a contributing role in the etiology of the MetS.

The present study has also identified a possible socioeconomic gradient in the consumption of dietary fructose. The observed association between crowding index and lower intakes of natural fructose may, in part, reflect an economic obstacle to the adoption of healthier dietary habits and particularly the consumption of fruits and vegetables. In line with our findings, a previous study conducted in Lebanon showed a positive association between socioeconomic status and adherence to a Mediterranean dietary style [75]. Altogether, the study findings on the link between added fructose intake with the MetS, and the socio-demographic predictors of high fructose intake call for adequate public health interventions aimed at reducing the population’s intake of added sugar. This may require a multi-faceted approach that includes product reformulation to decrease sugar levels in processed foods, taxation strategies targeting high sugar foods and beverages, elimination of sugar subsidies, restricting and regulating the marketing of sugar-rich foods and beverages, setting standards for food and drink offered in government-sponsored institutions, and launching awareness campaigns on the adverse impacts of sugar [83].

The findings of this study should be considered in light of the following limitations. First, the results of this study cannot infer causality given its cross-sectional design. Hence, the study findings are reflective of associational relationship between exposure (fructose intake) and outcome (MetS) and cannot be used to document a cause–effect relationship. However, in order to decrease possible reverse causation, participants who reported the previous diagnosis of a chronic disease or metabolic abnormalities that may have impacted their food consumption habits, were excluded from the study. Second, dietary assessment was based on the use of an FFQ, which may be limited by measurement errors, reliance on memory and the number of food items included in the food list [84]. Despite its potential limitations, the FFQ method has been shown to be one of the most suitable dietary assessment tools in epidemiological studies since it provides information on the subjects’ habitual diet over longer periods of time [85]. However, as is the case with most questionnaire-based surveys, the interview-based approach that was adopted in our study, may incur a social desirability bias, whereby subjects may respond in a manner that they perceive as more acceptable or favorable to the interviewer [86]. This may be particularly true for questions pertinent to smoking (which may get under-reported) [87] and physical activity (which may get over-reported) [88], a fact that may explain the lack of significant differences in these lifestyle practices, between those with and those without the MetS in our study. It is however important to note that, in our study, all personnel who conducted data collection had received extensive training in order to decrease judgmental verbal and nonverbal communication and minimize any social desirability bias. In addition, the FFQ and the IPAQ that were used in the present study were not validated in our population; however, these assessment tools were previously adopted in several dietary and physical assessment studies amongst Lebanese adults and yielded plausible associations with obesity, MetS, diabetes and metabolic abnormalities [34, 44, 53, 77, 89–94]. Finally, the present study was limited to the urban setting of the Greater Beirut area, and therefore findings pertinent to the consumption levels of foods, and hence fructose, may not be representative of less urban settings in the country [36]. The choice of Beirut for the study may be explained by the fact that it encompasses approximately 40% of the Lebanese population and is usually considered the melting pot of the country. Future studies, that are longitudinal in nature, and that include a larger and more diverse sample are needed to further confirm the results of this study and better elucidate the link between fructose and metabolic health.

## Conclusion

In conclusion, to our knowledge, this study is the first in the Arab Middle-East to assess fructose intake and investigate its association with MetS. The current study documented a high consumption level of dietary fructose amongst Lebanese urban adults, while also showing that, unlike natural fructose intake, high intakes of added fructose were associated with a three-fold increase in the odds of MetS. These findings may be used for the development of evidence-based interventions and public health strategies aimed at liming the consumption of added sugar from processed foods and encouraging healthier dietary patterns in the Lebanese population. Building on the demographic and socioeconomic factors that were investigated in this study, the planned public health interventions may particularly focus on the population subgroups that are more likely to consume excessive intakes of added sugar.

## Data Availability

The datasets used and/or analyzed during the current study are available from the corresponding author on reasonable request.

## Abbreviations

AOR: Adjusted Odds Ratio
AUB: American University of Beirut
BMI: Body Mass Index
BP: Blood Pressure
BPA: Bisphenol A
CHO: Carbohydrates
CI: Confidence Interval
EI: Energy Intake
EMR: Eastern Mediterranean Region
FBG: Fasting Blood Glucose
FFQ: Food Frequency Questionnaire
HDL-C: High-Density Lipoprotein Cholesterol
HFCS: High Fructose Corn Syrup
IDF: International Diabetes Federation
IPAQ: International Physical Activity Questionnaire
LDL-C: Low-Density Lipoprotein Cholesterol
MetS: Metabolic Syndrome
NCDs: Non-Communicable Diseases
NFSC: Nutrition and Food Sciences
NHANES: National Health and Nutrition Examination Survey
OR: Odds Ratio
RR: Relative risk
SPSS: Statistical Analysis Package for Social Sciences
SSBs: Sugar-Sweetened Beverages
TG: Triglycerides
USDA: United States Department of Agriculture
WC: Waist Circumference
11β-HSD1: 11β-Hydroxysteroid dehydrogenase 1
